# Participation and social functioning in patients with fibromyalgia: development and testing of a new questionnaire

**DOI:** 10.1186/1477-7525-11-135

**Published:** 2013-08-05

**Authors:** Erik Farin, Antje Ullrich, Johannes Hauer

**Affiliations:** 1 Department of Quality Management and Social Medicine, University Freiburg-Medical Center, Engelbergerstr 21, Freiburg D-79106, Germany

**Keywords:** Fibromyalgia syndrome, Social participation, Outcome assessment (Health Care), Rehabilitation

## Abstract

**Background:**

While there are numerous instruments for capturing the symptoms of fibromyalgia syndrome (FMS) patients, there is a lack of questionnaires capable of measuring in detail FMS patients’ participation and social functioning. It was our aim to develop and methodologically test a new patient questionnaire specific to FMS measuring these concepts (the “Fibromyalgia Participation Questionnaire” FPQ).

**Methods:**

We first conducted a qualitative prestudy (focus groups, N = 38) to identify which impairments FMS patients experience in daily life because of their illness. To analyze the data we developed a coding system that contained 10 supercategories and a total of 105 subcategories. Items for the FPQ were developed from the subcategories. The psychometric analysis was done on a sample of N = 256 FMS patients undergoing inpatient rehabilitation in Germany.

**Results:**

The final version of the FPQ contained 27 items and three scales (participation in social life FPQ-S, 11 items; participation in daily life FPQ-D, 11 items, participation in work-life FPQ-W 5 items). The FPQ displays good distribution properties, all the scales are unidimensional, and the scales fit to the Rasch model. Cronbach’s Alpha range from 0.85 to 0.94. We noted indications of construct validity in that the FPQ correlates as expected with the Fibromyalgia Impact Questionnaire (physical scale), Pain Disability Index and scales from the PROMIS® item banks for satisfaction with participation. The FPQ scales generally reveal greater responsiveness than other instruments. By linking FPQ items to the categories of the International Classification of Functioning, Disability and Health (ICF) we demonstrate content validity.

**Conclusions:**

The FPQ captures participation and social functioning in FMS patients. As its psychometric properties are good, it can be recommended for use in evaluation studies and clinical trials.

## Background

Fibromyalgia (FMS) is a syndrome of unknown etiology characterized by chronic pain, decreased pain threshold or tender points, fatigue, disturbed sleep, stiffness, cognitive dysfunction, anxiety and depression [1,2]. These symptoms considerably impair the activities and social participation of FMS patients [3-5] and ultimately result in a lower health-related quality of life [6,7]. Increasing effort has recently been made to determine the full impact and wider influence of FMS and other musculoskeletal diseases and to measure the personal and social impact captured by participation and social functioning (i.e., [8-10]). Under certain circumstances, the social consequences of FMS (such as problems at work, or difficulties getting together with friends) can often be even more challenging than the limitations patients experience with body functions and activities (i.e., pain, difficulty walking longer distances).

While there are numerous instruments for capturing the symptoms of FMS patients, (with [11,12] providing a fine overview), there is a lack of questionnaires capable of measuring in detail FMS patients’ participation and social functioning. The Fibromyalgia Impact Questionnaire (FIQ/FIQ-R [13,14]) is the only disease-specific instrument currently available (see [15]). However, it focuses closely on body functions and elementary activities within the domain of domestic life [15]. More complex aspects of participation such as interpersonal interactions, social relationships, work and employment are either not addressed at all or if so, with very few items (vgl. [16]). Furthermore, the lack of evidence for the instrument’s factorial validity has been criticized [17], and there are claims of sex and ethnicity biases [12].

Prodinger et al. [16] report on testing a clinical instrument to measure functioning in FMS patients with categories from the International Classification of Functioning, Disability and Health (ICF) covering body functions, body structures, activity and participation. Yet the items addressing activity and participation employ the existing ICF-Core-Set for Chronic Widespread Pain, and were developed without specifically addressing FMS patients, moreover, important categories were eliminated in the psychometric testing (i.e., d920 recreation and leisure).

Van Eijk-Hustings et al. [18] measure participation via time spent on unpaid tasks, paid work, chores, leisure activities and social activities. It is questionable as to whether the mere number of hours is a valid parameter for measuring participation restriction, as this perspective disregards subjectively-experienced difficulties associated with social activities and context factors (such as family and professional obligations). Wilkie et al. [8] outline further measures of social function and participation in musculoskeletal populations and their pros and cons. Only one instrument (the generic questionnaire “Impact on Participation and Autonomy” [19]) has been administered to FMS patients. In light of the current situation concerning outcome measurements of FMS patients, Arnold et al. ([5], p. 119) conclude that “improved functional and quality of life measures for fibromyalgia are clearly needed”.

To summarize: there is no disease-specific instrument to measure the participation and social functioning of FMS patients in particular that has also been psychometrically validated. Our study’s aim was therefore to develop and test such an instrument (the “Fibromyalgia Participation Questionnaire” FPQ). Such a tool would make available a fibromyalgia-specific instrument for therapy evaluation and clinical studies with which the patients’ important outcome variables, namely participation and social functioning, can be captured. By involving FMS patients in the developmental process and by considering their specific problems, one can expect that this instrument will cover areas particularly relevant to the patients, and that it possesses high sensitivity to change. Its diagnostic application when treating FMS patients would be appropriate when the physician desires an overall impression of the everyday difficulties associated with FMS at the beginning of treatment. This information may prove relevant to the subsequent therapy (i.e., the urgency of psychological or psychosocial measures), and it cannot always be systematically captured via face-to-face consultation with the patient alone.

## Methods

### Instrument development - focus groups and cognitive interviews

To determine the specific participation restrictions FMS patients experience it is worthwhile questioning patients about how they personally experience problems employing qualitative methodology (cf. [5,20]). In a pre study we examined eight focus groups (N = 38), each containing three to six FMS patients [21]. The pre study was conducted in September and October 2010. The interviews lasted between 60 and 90 minutes and were audio-recorded in digital format. In line with Anatchkova & Bjorner [22], we had two data-collection phases: in the first, we used a half-structured interview guideline, asking the patients to freely describe the participation restrictions they experience from their FMS on a daily basis. The key questions were: ‘In which areas of your daily life does your illness hamper you?’ and ‘How important to you are those areas in your daily life?’ The second phase contained activating elements [23]. Using colored index cards, we illustrated eight areas of participation (covering chapters 6 to 9 in the ICF) with examples, and the patients were to select three areas in which their illness hampered them the most. We thereby tested the relevance of the areas of participation we had theorized that the patients would experience. Once the patients had selected the index cards, the results were discussed in a focus group.

The group discussions were recorded and transcribed, and the contents analyzed by two coders using Atlas.ti software (version 6.2). A coding system was developed in several steps. The final version contained 10 supercategories (i.e., “social activities/recreation/leisure”) and a total of 105 subcategories (e.g., “going to a concert”). The patients experienced especially strong impairment in the following areas of daily life: contact with close family and friends, grandchildren, colleagues, partners, during leisure activities, at work, and when doing housework (for more detail, see [21]). 69 items were generated from the subcategories while we made sure that the contents of the most frequently-coded subcategories were also adequately represented in the items. Of the 69 items (=FPQ version 1), 11 address work and job-training and are only to be answered when the subject had answered “yes” to the question “Are you employed or in a job-training program (i.e., school, apprenticeship, higher education)?”.

The FPQ version 1 was presented to 10 FMS patients in a cognitive interview [24] in which thinking-aloud and verbal-probing techniques were used. The interviews were conducted in May 2011. To reduce the patients’ burden, the FPQ was subdivided into two approximately equally long versions A and B. Five patients were given Version A and five Version B. Their remarks were used to revise and if necessary, omit items. After the cognitive interviews, 47 items remained (FPQ version 2). Of those 47 items, 9 address employment and training. The FPQ contains questions (such as “To what extent did you have difficulty engaging in your hobbies during the last four weeks because of your illness?”) for which there were six possible response categories (no difficulty, mild difficulty, moderate difficulty, severe difficulty, impossible, not applicable).

**Figure 1:**
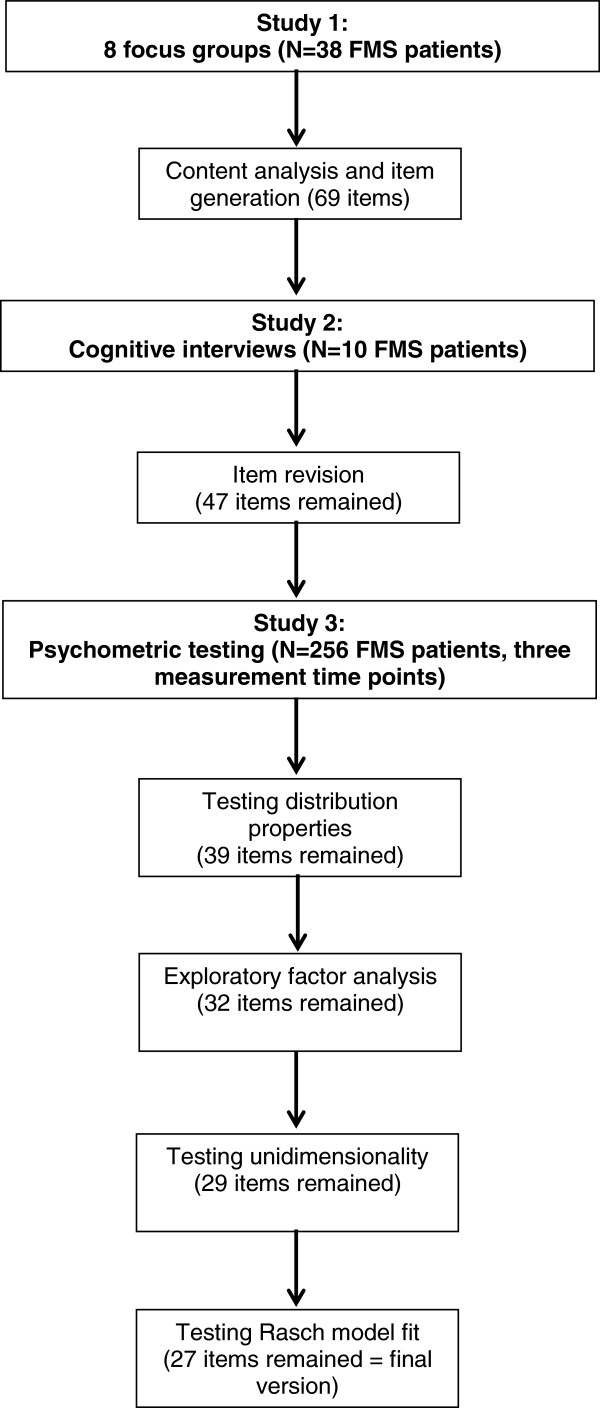
Study design and course of study.

A summary of the entire study design – from instrument development to psychometric testing – is presented in Figure 1.

### Sample

To test the FPQ psychometrically, N = 256 FMS patients undergoing inpatient rehabilitation in Germany were surveyed at the beginning of rehabilitation (t0), 3 months (t1) and 6 months (t2) after the end of rehabilitation. The recruitment phase was between August 2011 and September 2012. An interdisciplinary treatment program was carried out during rehabilitation that comprised coordinated psychological, medical, pharmacologic, educational, and physiotherapeutic components. The study’s inclusion criterion was a definitive diagnosis of “fibromyalgia syndrome” (ICD-10; M 79.7). FMS was diagnosed according to the American College of Rheumatology (ACR) guidelines. The study was approved by the ethics committee of the University of Freiburg (approval number 172/10). The percentage of patients that did not fill out the questionnaire was 35.4%. The most important reason for non-inclusion was refusal to participate (70.9%) followed by language difficulties (11.9%). Table 1 provides information on the study patients. The return rate at t1 was 83.6%, and 72.3% at t2. The dropout analysis revealed that the participants at t2 did not differ significantly from dropouts regarding education, gender, and chronification, as well as the FPQ and FIQ values. Only age made a significant difference. The dropout patients were somewhat older (53.4 versus 50.7 years, p = 0.019). We referred to t0 data in all the analyses, adding t1 and t2 data only to determine the responsiveness.

**Table 1:** **Respondent characteristics** (**N** = **256**)

	
**Age** (Mean/SD)	52.6 (8.4)
**Sex**	
% Female	91.3
**Level of education** (highest level completed, %)	
Elementary school	30.9
Secondary school	42.6
University-entrance diploma or technical college qualification	24.1
**Employment**	
% Employed	80.3
**Chronification** (%)	
< 1 year	2.4
1-2 years	4.8
2-5 years	13.5
5-10 years	20.7
>10 years	53.4
**Perceived health status** (%)	
Very good	0
Good	2.4
Satisfactory	7.6
Not so good	36.8
Bad	41.6
Very bad	11.6
**Pain impact**	
(FIQ-G, mean/SD)	52.4 (12.9)
**Anxiety and depression**	
(HADS, mean/SD)	
Anxiety	10.8 (4.0)
Depression	9.4 (4.5)

With regard to the FIQ-G, higher value means higher disease impact (range 0–80); with regard to the HADS, higher values mean higher level of anxiety or depression (values ≤ 7: inconspicuous, values between 8–10: borderline, values ≥ 11: conspicuous).

*SD* standard deviation.

### Instruments

In addition to the FPQ version 2, we administered three other instruments to test construct validity and responsiveness, and to compare the FPQ’s contents with other instruments. The FIQ’s physical scale (German version FIQ-G [25]) contains 10 items and approaches the construct participation and social functioning in that it addresses key daily tasks (i.e., going shopping) and because one item refers to visiting friends and relatives. The Pain Disability Index (PDI [26]; German version: [27]) is a generic measure of pain-related disability that can be used for pain in all body regions. The short instrument contains 7 items addressing participation restrictions in various fields of life (i.e. relaxation, social activities, work) and has been administered often in FMS patients [28,29]).

The PROMIS® item banks for satisfaction with participation are generic instruments that capture participation and social functioning (differentiating between social roles (SR) and discretionary social activities (DSA)). The two item banks are components of the PROMIS® social health domain framework [30,31]. Our research group received permission from the PROMIS network to translate the item banks regarding satisfaction with participation into German, and the results of the psychometric assessment in patients with chronic back pain will be published soon [32]. Results of the final 13-item (SR-G) and 10-item (DSA-G) German scales regarding unidimensionality were satisfactory. Both scales are reliable and show good item response model fit and distribution characteristics. Both scales are sensitive to clinical changes, and we observed initial proof of construct validity [32].

### Analyses

#### Psychometric analyses

The sequence of psychometric analyses was based on the procedure described by Reeve et al. [33] and Rose et al. [34]. For our analyses we applied IBM SPSS Statistics (Version 20), IBM SPSS AMOS (Version 20) and WINSTEPS (Version 3.68) software programs.

Response frequency and ceiling/floor effects: An item was removed if one of these conditions was fulfilled: a) more than 15% missing values (missing or “not applicable”), or b) ceiling or floor effects (more than 50% of values in the extreme categories).

Exploratory factor analysis: To determine the number of factors to be extracted, the following criteria were used: a) scree test, b) interpretability, and c) explained variance. An item was removed if it did not load unambiguously on the extracted factors (factor loading > =0.50 on exactly one factor; factor loading < =0.50 on all other factors). The 9 work-related items were analyzed separately from the rest, as they were only answered by a limited number of respondents.

Unidimensionality and local independence: Every scale was tested separately to check whether it measures a latent dimension. Single-factor confirmatory factor analyses were carried out. Model fit was evaluated using the Comparative Fit Index (CFI) [35], the Tucker-Lewis Index (TLI) [36], the root mean square error of approximation (RMSEA) and the standardized root mean square residual (SRMR). CFI and TLI values >0.90 are an indication of good fit. RMSEA values < 0.10 suggest moderate fit; values < 0.05 are a good fit. The SRMR value should be under 0.08 [35]. Unidimensionality is assumed whenever at least three of four parameters produce good values. Covariances between error terms were set free only if theoretically justifiable. The analysis of local independence examines the residual correlation matrix produced by the single-factor CFA. The absolute values of the residual correlations should all be lower than 0.20, and the proportion of residual correlations under 0.10 should be as small as possible (see [33]).

IRT analyses: The 1-parameter IRT model (Rasch model [37]) was used. Infit and outfit mean square statistics (Infit MNSQ, Outfit MNSQ) were applied as goodness-of-fit statistics. Poor item fit was defined as infit or outfit <0.6 or >1.4 ([37], p. 179). Items with a poor infit or outfit were eliminated.

#### Reliability

To determine reliability, Cronbach’s Alpha and the person separation index (PSEP) were calculated. PSEP describes the number of performance levels the test measures in a particular sample; it can be converted to a reliability value [38].

Differential item functioning (DIF): DIF was tested in reference to age, gender and education. We used ordinal logistic regression models to evaluate DIF [39]. To determine the size of the DIF, we determined the increase in Nagelkerkes R^2^ after including the DIF variable and DIF variable-sum score interaction. A value greater than 0.03 was considered a criterion for noticeable DIF (see [34]).

#### Responsiveness

Internal Responsiveness characterizes the ability of a measure to change over a particular time frame. We calculated standardized response means (SRM). As in Cohen [40], values of 0.20 were considered “small”, around 0.50 “medium”, and > 0.80 were deemed “large”.

#### Construct validity and content validity

To prove construct validity, we tested the following hypothesis: We would expect to observe positive and large (r > 0.50) correlations between the FPQ on the one hand, and with the FIQ-G (physical scale), PDI, SR-G and DSA-G on the other. The more similar the scales’ contents are, the closer these relationships should be.

## Results

### Psychometric analyses

We eliminated eight items because of too many missing values. These were generally questions that addressed a participation area that was irrelevant to some individuals (i.e., gardening, activities with grandchildren, dancing, etc.). The subsequent exploratory factor analysis revealed a two-factor solution for the non-job-related items that can be interpreted as: Factor 1: Participation in social life (FPQ-S), Factor 2: Participation in daily life (FPQ-D). The job-related items showed a one-factor solution (participation in work-life FPQ-W).

After the exploratory factor analysis, 32 items remained. To ensure sufficient unidimensionality, three items in the FPQ-S scale were eliminated. No items needed to be eliminated from the other two scales. To ensure the fit to the Rasch model, one item in the FPQ-S scale and one item in the FPQ-W scale had to be eliminated. The final version of the FPQ questionnaire thus contained 27 items (FPQ-S: 11 items, FPQ-D: 11 items, FPQ-W: 5 items). The scale values lie between 0 and 100, with the higher values corresponding to better participation and social functioning.

Table 2 illustrates all FPQ’s psychometric properties. The instrument displays good distribution properties, all the scales are unidimensional and there is only minor local dependence. The FPQ-S and FPQ-D scales fit to the Rasch model very well, the FPQ-W scale’s fit is satisfactory (one item’s outfit value is at 1.46 somewhat too high, that of another at 0.50 too low). The fit to the Rasch model guarantees some methodological advantages [37,41]: The FPQ person parameters are interval scale measures, person parameters can (as in adaptive testing) also be derived from a portion of the scale items, and the person fit can be analyzed so that inattentive responders or cognitively-challenged patients can be identified. The reliability values are good to very good (with Cronbach’s Alpha ranging from 0.85 and 0.94); none of the items reveals differential item functioning. The corrected item-scale correlations are generally above 0.60 (see Table 3).

**Table 2:** Psychometric properties of the FPQ scales

	**Participation in social life FPQ-S (11 items)**	**Participation in daily life FPQ-D (11 items)**	**Participation in work-life FPQ-W**^**1 **^**(5 items)**
**Distribution properties**			
Mean percentage of missing values and ‘not applicable’ (%)	8.1	4.3	4.1
Scale mean (sum score, 0–100, 100 = best possible participation) (mean, standard deviation, N)	39.2 (21.2) N = 223	60.3 (20.6) N = 239	34.6 (21.3) N = 184
**Unidimensionality**			
Fit values	CFI = 0.943	CFI = 0.944	CFI = 0.985
	TLI = 0.929	TLI = 0.930	TLI = 0.969
	RMSEA = 0.099	RMSEA = 0.089	RMSEA = 0.106
	SRMR = 0.042	SRMR = 0.046	SRMR = 0.025
**Local independence**			
Percentage of residual correlations >0.10 (%)	9.1	7.3	30.0
Percentage of residual correlations >0.20 (%)	0	0	0
**IRT analysis**			
Number of items with poor item fit (infit or outfit <0.6 or >1.4)	0	0	2
**Reliability**			
Cronbach’s Alpha	0.94	0.93	0.85
Person separation index	0.91	0.90	0.91
**Differential item functioning**			
Number of items with DIF	0	0	0

1 only for persons employed outside the home or in job training (i.e., school, apprenticeship, college).

**Table 3:** **Items in the FPQ questionnaire** (**in order of mean values within the scales**)

**Item (item code)**	**Mean (Standard deviation)**	**Corrected item-scale correlation**
**Participation in social life FPQ-S (11 items)**	**39.2 (21.2)**	
Organizing a party for friends or family? (23)	3.82 (1.06)	0.76
To handle the day after a very busy day as well as usual (22)	3.78 (0.87)	0.62
Undertaking city trips ? (11)	3.59 (1.22)	0.76
Inviting several friends to your flat or home (10)	3.52 (1.17)	0.81
Coping with your daily routine (such as job, housework, private life) (25)	3.51 (0.89)	0.62
Going out in the evening? (31)	3.49 (1.11)	0.82
Managing to balance your private life with your job and/or housework responsibilities? (19)	3.41 (1.08)	0.69
Engaging in your hobbies? (17)	3.35 (1.03)	0.70
Attending parties or celebrations with lots of guests? (09)	3.23 (1.24)	0.76
Attending cultural events (i.e., concerts, the cinema, theatre)? (15)	3.10 (1.27)	0.82
Doing things with your partner? (27)	2.89 (1.01)	0.72
**Participation in daily life FPQ-S (11 items)**	**60**.**3 (20**.**6)**	
Entertaining an unannounced guest? (35)	3.10 (1.16)	0.64
Organising your private life well ? (33)	2.86 (0.99)	0.77
Taking care of necessary paper work (i.e., insurance policies, tax forms, financial records) (18)	2.84 (1.19)	0.70
Getting the shopping done ? (32)	2.81 (0.99)	0.71
Making sound decisions in daily life? (34)	2.80 (1.11)	0.74
Keeping your usual appointments? (12)	2.74 (1.06)	0.71
Keeping dates with friends? (24)	2.74 (1.13)	0.72
Going out to eat in your free time? (06)	2.49 (1.14)	0.74
Making telephone calls? (28)	2.24 (1.09)	0.67
Going to the bank to take out money or pay bills? (37)	2.05 (1.09)	0.72
When paying for something taking your change or handing over the cash (i.e., when shopping)? (07)	1.77 (0.99)	0.59
**Participation in work**-**life FPQ-W (5 items)**	**34**.**6 (21**.**3)**	
Carrying out several duties simultaneously? (43)	3.69 (1.09)	0.74
Performing your usual tasks at work (i.e., carrying things, working at the computer, etc.?) (39)	3.65 (0.85)	0.66
Doing your job at the necessary speed? (41)	3.61 (1.08)	0.83
Concentrating on the job at hand when at work? (42)	3.59 (0.91)	0.72
Meeting the demands made upon you while at work? (44)	3.52 (1.09)	0.89

**Instructions**: To what extent has your illness made it difficult for you to carry out the activities listed below during the last 4 weeks?

**Response categories are**: 1 = no difficulty, 2 = mild difficulty, 3 = moderate difficulty, 4 = severe difficulty, 5 = impossible.

**Scale values**: Range is 0–100, higher values indicate better participation and social functioning.

### Descriptive results

Table 3 shows descriptive results on the scale and item level. The scale values illustrate considerable participation restrictions in social interactions and activities in the FPQ-S area as well as in the professional area (FPQ-W) (mean values ranging from 35 to 40). In the FPQ-D scale (daily tasks and responsibilities) we noted considerably fewer participation restrictions (mean value approx. 60). FMS patients have particular difficulty socializing, i.e. going to parties or accepting invitations, going on vacation, and generally with leisure activities and attending cultural events. At work they seem to have trouble coordinating multiple duties at the same time and maintaining the necessary working tempo.

### Construct validity

Table 4 illustrates that we proved our hypothesis. The FPQ correlates closely with the four other scales that also capture facets of participation and social functioning. The FPQ-S and FPQ-D scales correlate higher than 0.60 with the other instruments, while the job-related FPQ-W scale correlates at between 0.44 and 0.59. This is most probably because job-related issues are not addressed at all in the other scales and if they are, they are captured only through a few items.

**Table 4:** Correlations between FPQ scales and other instruments assessing aspects of participation and social functioning

	**FIQ-G (Physical scale)**	**PDI**	**Satisfaction with participation in social roles (SR-G)**	**Satisfaction with participation in discretionary social activities (DSA-G)**
**Participation in social life FPQ**-**S**	−0.69	−0.70	0.67	0.70
**Participation in daily life FPQ**-**D**	−0.70	−0.65	0.66	0.62
**Participation in work**-**life FPQ**-**W**	−0.45	−0.59	0.57	0.44

In the FPQ, SR-G and DSA-G, high values reflect high participation values, while in the FIQ-G and PDI, low values stand for high participation. Pearson correlations, N_min_ = 172, N_max_ = 235, p < .001 for all correlations.

### Responsiveness

To investigate any differences between the FPQ and other instruments in terms of responsiveness, Table 5 shows the effect sizes 3 and 6 months after rehabilitation, categorized by subgroups. One notes that moderate effects were achieved over the short term (3 months after rehabilitation) that diminished 6 months after rehabilitation. The highest responsiveness was revealed by the FPQ scales (especially the FPQ-W) and the PDI, followed by the SR-G scale. SRM values were lower in the FIQ-G and DSA-G scales.

**Table 5:** **Responsiveness of FPQ scales and other instruments in subgroups** (**standardized response mean**)

	**Participation in social life FPQ-S**		**Participation in daily life FPQ**-**D**		**Participation in work**-**life FPQ**-**W**		**FIQ**-**G (Physical scale)**		**PDI**		**Satisfaction with participation in social roles (SR-G)**		**Satisfaction with partici**-**pation in discretionary social activities (DSA-G)**	
	**3 m**	**6 m**	**3 m**	**6 m**	**3 m**	**6 m**	**3 m**	**6 m**	**3 m**	**6 m**	**3 m**	**6 m**	**3 m**	**6 m**
**Total**	**0**.**47**	0.27	**0**.**45**	0.28	**0**.**52**	**0**.**42**	0.37	0.18	**0**.**53**	0.37	**0**.**45**	0.19	0.30	0.18
**Sex**														
Male (N_max_ = 19)	0.20	0.16	0.36	0.33	**0**.**79**	**0**.**44**	**0**.**55**	**0**.**60**	0.12	**0**.**54**	0.37	**0**.**48**	**0**.**60**	**0**.**56**
Female (N_max_ = 192)	**0**.**49**	0.27	**0**.**46**	0.27	**0**.**49**	**0**.**42**	0.35	0.14	**0**.**57**	0.37	**0**.**47**	0.16	0.28	0.14
**Age**														
<50 years (N_max_ = 71)	0.33	0.32	0.35	0.34	**0**.**48**	**0**.**65**	0.25	0.05	**0**.**60**	**0**.**57**	**0**.**46**	0.22	0.39	**0**.**49**
50-55 years (N_max_ = 59)	**0**.**61**	0.30	**0**.**57**	0.19	**0**.**78**	0.25	**0**.**56**	0.34	**0**.**55**	0.34	**0**.**48**	0.11	0.26	0.23
>55 years (N_max_ = 82)	**0**.**47**	0.17	**0**.**45**	0.28	0.37	0.33	0.32	0.22	**0**.**42**	0.23	**0**.**42**	0.22	0.22	0.14
**Education**														
Elementary school (N_max_ = 70)	**0**.**50**	**0**.**42**	**0**.**40**	**0**.**41**	**0**.**43**	0.30	0.38	0.22	**0**.**41**	0.31	0.37	0.21	0.31	0.31
Secondary school (N_max_ = 85)	**0**.**48**	0.13	**0**.**56**	0.17	**0**.**60**	**0**.**47**	0.35	0.07	**0**.**69**	0.26	**0**.**43**	0.09	0.31	0.06
University-entrance diploma or technical college qualification (N_max_ = 48)	0.39	0.25	**0**.**42**	0.24	**0**.**54**	**0**.**45**	0.33	0.21	**0**.**43**	**0**.**53**	**0**.**49**	0.16	0.29	0.20
**Chronification**														
<5 years (N_max_ = 43)	**0**.**46**	**0**.**44**	0.35	**0**.**61**	**0**.**65**	**0**.**87**	0.06	0.25	**0**.**54**	**0**.**72**	0.15	0.33	0.11	0.20
5-10 years (N_max_ = 39)	**0**.**48**	0.04	**0**.**47**	−0.04	**0**.**57**	0.35	**0**.**47**	0.15	**0**.**68**	0.30	**0**.**89**	0.26	**0**.**53**	0.15
>10 years (N_max_ = 115)	**0**.**40**	0.21	**0**.**49**	0.25	**0**.**46**	0.24	**0**.**44**	0.14	**0**.**48**	0.25	**0**.**42**	0.03	0.25	0.08

3 m/6 m: 3 months/6 months after end of rehabilitation; values > =0.40 are printed in bold.

Our subgroup analyses revealed that the FPQ shows effects in all the subgroups we considered. The effects are less pronounced 6 months after rehabilitation in older persons and in those whose illness is of longer duration. As the other instruments tend to reflect such results also, we cannot claim to have demonstrated group-specific FPQ responsiveness, but rather the influence of general risk factors on the success of interdisciplinary treatment programs in FMS patients.

### Content validity

To determine whether the FPQ covers relevant areas of participation, and the extent to which these differ from those addressed in the other instruments, we linked items in all the instruments to the ICF categories (Table 6). We chose specific and less specific ICF categories according to how specific or general the content was in a given item. We observed that no other instrument contains items from so many different ICF chapters of activity and participation (7 of 9 chapters) as do the three FPQ scales. The PDI measures at a similar breadth (5 of 9 chapters). However, the FIQ (physical scale), SR-G, and DSA-G each contains only items from three ICF chapters. In contrast to the other instruments, the FPQ focuses strongly on the general tasks and demands areas (i.e., “managing your general daily routine (job, housework, private life)”) as well as on community, social and civic life - areas that no other instrument addresses at such a high rate (30% of items). The FPQ is furthermore the only instrument containing a specific scale for participation restrictions addressing work and employment (see [5]). Although the SR-G scale does cover this area with several items, the items do not specify whether the patient is describing housework or paid employment (thus explaining the double allocation in Table 6).

**Table 6:** **Linking of the items of the FPQ scales**, **FIQ**-**G** (**physical scale**), **PDI**, **SR**-**G and DSA**-**G to the ICF**

	**FPQ-S**	**FPQ-D**	**FPQ-W**	**FIQ-G (physical scale)**	**PDI**	**SR**-**G**	**DSA**-**G**
**Body functions**					PDI7		
**Activities and participation**							
**Chapter 1 Learning and applying knowledge**							
Focusing attention (d160)			*FPQ*-*W42*				
**Chapter 2 General tasks and demands**							
Undertaking multiple tasks (d220)			*FPQ*-*W43*				
Carrying out daily routine (d230)	FPQ-S22, S25	FPQ-D33, D34, D12, D24					
**Chapter 3 Communication**							
Using communication devices and techniques (d360)		FPQ-D28					
**Chapter 4 Mobility**							
Walking (d450)				FIQ7			
Driving motorized vehicles (d4751)				FIQ10			
**Chapter 5 Self**-**care**					PDI6		
**Chapter 6 Domestic life**					PDI1	*PROMIS*-*SR1*,*SR3*, *SR4*,*SR6*, *SR7*	
PROMIS-SR8,SR9, SR10, SR11							
Acquisition of goods and services (d620)		FPQ-D32		FIQ1		PROMIS-SR13	
Household tasks (d630-d649)		FPQ-D35		FIQ2 - FIQ6			
Caring for household objects (d650)				FIQ9			
Assisting others (d660)						*PROMIS*-*SR2*,*SR5*,*SR12*	*PROMIS*-*DSA3*, *DSA7*, *DSA8*
**Chapter 7 Interpersonal interactions and relationships**							
General interpersonal interactions (d710-d729)	FPQ-S10				PDI3		PROMIS-DSA1, DSA5, DSA9
*PROMIS*-*DSA3*, *DSA7*, *DSA8*							
Informal social relationships (d750)							PROMIS-DSA2
Family relationships (d760)	*FPQ*-*S23*					*PROMIS*-*SR2*,*SR5*,*SR12*	
Intimate relationships (d770)	FPQ-S27				PDI5		
**Chapter 8 Major life areas**							
Work and employment (d840-d859)			*FPQ*-*W43 W42*, FPQ-W39, W41,W44		PDI4	*PROMIS*-*SR1*,*SR3*, *SR4*,*SR6*, *SR7*	
Basic economic transactions (d860)		FPQ-D07					
Complex economic transactions (d865)		FPQ-D18, D37					
**Chapter 9 Community, social and civic life**							PROMIS-DSA6
Ceremonies (d9102)	*FPQ*-*S23*, S09						
	**FPQ-S**	**FPQ-D**	**FPQ-W**	**FIQ-G (physical scale)**	**PDI**	**SR**-**G**	**DSA**-**G**
Recreation and leisure (d920)	FPQ-S11,S31, S19	FPQ-D06			PDI2		PROMIS-DSA4, DSA10
Arts and culture (d9202)	FPQ-S15						
Hobbies (d9204)	FPQ-S17						
Socializing (d9205)				FIQ8			

Items have been linked to an ICF category as specific as possible; italics = double assignment. In linking the FIQ-G’s items, we applied results from Prodinger et al. [15]. The assignment of the FPQ item codes to the complete item wording can be found in Table 3. FPQ-S = FPQ Participation in social life, FPQ-D = FPQ Participation in daily life, FPQ-W = FPQ Participation in work-life, SR-G = Satisfaction with participation in social roles (derived from PROMIS® item bank), DSA-G = Satisfaction with participation in discretionary social activities (derived from PROMIS® item bank).

## Discussion

The FPQ’s psychometric properties are good. We provide evidence of unidimensionality, local independence, reliability, Rasch-model fit, absence of differential item functioning, responsiveness, and construct validity. We believe that the FPQ has advantages over other instruments assessing participation and social functioning. An advantage over the disease-specific FIQ is that the FPQ more strongly captures relevant participation domains such as contact with close friends and family members, free-time and leisure activities, and one’s profession. The importance of interpersonal relationships to the quality of life of FMS patients is revealed in our focus group study and other research efforts [42]. The FPQ also covers the ICF chapters of domain activity and participation more thoroughly than the FIQ. These advantages are manifested to an even greater degree when compared with the revised version (not employed here) (FIQ-R, [14]), as when revising the physical FIQ-R scale, the only item allocated to social contacts (“visit friends/relatives”) was eliminated.

Other advantages of the FPQ over the FIQ’s physical scale are the former’s greater responsiveness (proved in the short and long term) as well as the FPQ’s thorough psychometric examination, its Rasch-model fit, and the absence of differential item functioning. As far as we know, these psychometric properties have not yet been demonstrated for the FIQ and FIQ-R. Our instrument’s greater responsiveness compared with the FIQ is even more cogent because of evidence that the FIQ is more responsive than outcome measures like patient ratings of pain intensity or total tender point pain intensity [43].

However, the FPQ captures only participation and social functioning and not the overall impact of fibromyalgia. Thus, it presents no appropriate alternative to the FIQ for many study efforts. The combined use of both instruments is conceivable in cases, where both the overall impact and the participation and social functioning are to be measured. The correlations between the FPQ and FIQ (0.45 to 0.70, Table 4) demonstrate that related but nevertheless distinct constructs are being assessed.

Another advantage of the FPQ over generic participation and social functioning instruments (i.e., the PROMIS® item bank, the PDI or others presented by Wilkie et al. [8], Eyssen et al. [44] or Noonan et al. [45]) is the FPQ’s specificity for FMS patients. All of its items are derived from FMS patient input in focus groups. Furthermore, of the existing generic instruments, few (with the PROMIS® item bank being the exception) have been subjected to such rigorous psychometric examination as the FPQ, and few contain a job-related scale capturing participation in work life. By developing a separate job-related scale with an introductory question addressing employment, a field of great importance to FMS patients [5,6,46,47] can be assessed in detail. The alternative to include work-related items in an overall scale has the disadvantage of many missing values as a relevant portion of FMS patients is unemployed.

The strengths of our study are that content validity has been guaranteed in qualitative prestudies, that we undertook thorough psychometric testing in a large cohort of FMS patients, and that both genders were recruited for the study. A study limitation is that these items were generated solely from results we gathered from focus groups and not through triangulation (i.e., additional in-depth interviews). Further limitations are that we had a relatively high percentage of non-responders and that we retrieved all our data from one particular healthcare setting (one inpatient rehabilitation center in Germany), meaning that further testing is needed to ensure our findings’ generalizability.

## Conclusion

With its three scales and 27 items, the FPQ measures participation and social functioning in FMS patients. As its psychometric properties including responsiveness are good, it can be recommended for use in evaluation studies and clinical trials. Future studies should determine psychometric properties in other settings and populations, examine re-test reliability, and analyze the FPQ’s advantages and disadvantages over generic participation instruments in greater depth.

## Abbreviations

CFI: Comparative fit index; DIF: Differential item functioning; DSA-G: Scale “Discretionary social activities” (German version of a scale based on PROMIS® item banks for satisfaction with participation); FIQ: Fibromyalgia impact questionnaire; FMS: Fibromyalgia syndrome; FPQ: Fibromyalgia participation questionnaire; ICF: International classification of functioning, disability and health; IRT: Item response theory; PSEP: Person separation index; RMSEA: Root mean square error of approximation; SR-G: Scale “Social roles” (German version of a scale based on PROMIS® item banks for satisfaction with participation); SRMR: Standardized root mean square residual; TLI: Tucker-Lewis Index.

## Competing interests

The authors declare that they have no competing interests.

## Authors’ contributions

EF defined the research theme; EF and AU designed research; AU, JH and EF conducted research; EF analyzed data; EF, AU and JH wrote the paper; EF had primary responsibility for final content. All authors read and approved the final manuscript.
